# Predictors of Cognitive Distortions in Intimate Partner Violence: An Analysis of Ideological, Relational and Sociodemographic Factors

**DOI:** 10.3390/bs15050677

**Published:** 2025-05-15

**Authors:** Patricia Medinilla-Tena, Marta Badenes-Sastre, Francisca Expósito

**Affiliations:** 1 Mind, Brain and Behavior Research Center, 18011 Granada, Spain; mbsastre@ugr.es (M.B.-S.); fexposit@ugr.es (F.E.); 2 Department of Social Psychology, University of Granada, 18011 Granada, Spain

**Keywords:** intimate partner violence, perceived severity, attributed self-responsibility, dependency, commitment, attitudes, ideology

## Abstract

This study explores how ideological, relational, and sociodemographic factors predict women’s perceptions of severity and self-responsibility across different types of intimate partner violence (IPV). Using an intra-subject design, 257 women completed the measures of interest, 191 experiencing IPV and 66 non-victims, according to their responses to WHO instruments. Participants were recruited via dissemination of the study through WhatsApp, Instagram, Facebook and institutional email. The results indicated that the primary predictors of perceived severity were ambivalent sexism in physical violence; favorable attitudes toward IPV (all forms of violence); feminist identity (physical violence and controlling behaviors); dependency (psychological violence); commitment (physical sexual, and controlling behaviors); education level (physical violence); and age (sexual violence). For attributed self-responsibility, ambivalent sexism and favorable attitudes toward IPV were predictors for controlling behaviors. Commitment and dependency, being a victim and age-influenced psychological violence, whereas being a victim predicted sexual violence. In addition, physical violence is perceived as the most severe and controlling behavior, eliciting greater levels of attributed self-responsibility than other forms of IPV. These results highlight the main predictors of cognitive distortions (perceived severity of violence and attributed self-responsibility), offering insight into the processes that victims in IPV situations undergo.

## 1. Introduction

Intimate partner violence (IPV) is one of the most common forms of violence against women, including physical, sexual, psychological, and controlling behaviors by a male partner or ex-partner (World Health Organization, 2024). IPV is one of the most extreme forms of inequality between men and women and occurs around the world (Carbonell Marqués & Mestre, 2019). This violence is devastating for women, causing injuries, unwanted pregnancies, abortions, sexual problems, depression, stress, anxiety, and a long list of other consequences (World Health Organization, 2024). It is estimated that approximately 30% of women worldwide experience the consequences of IPV (Spanish Ministry of Equality, 2020; World Health Organization, 2024), which prompts them to identify situations of violence and to adopt measures aimed at preserving women’s well-being and safety (Badenes-Sastre et al., 2025).

In recent years, the prevalence of IPV has undergone significant changes. Although physical violence continues to be perceived as the most severe and has the most serious consequences (Badenes-Sastre et al., 2023a; Wilson & Smirles, 2022), psychological violence has emerged as the most frequently reported form, closely followed by controlling behaviors (Medinilla-Tena et al., 2024; Spanish Ministry of Equality, 2020; Sánchez-Hernández et al., 2020). In this context, the incorporation of technologies has favored new manifestations of violence, such as digital control, that amplify the dynamics of power and control (Sarmiento, 2023). These dynamics remain rooted in the same patriarchal cultural structures that sustain unequal power relations (Sánchez-Hernández et al., 2020) and can make identification difficult. Therefore, considering all types of IPV manifestations will be fundamental to addressing it.

## 2. Perceived Severity of and Attributed Self-Responsibility in IPV

Perceived severity of IPV is defined as people’s subjective assessment of the magnitude or relevance of the threat, influencing decision-making (Badenes-Sastre et al., 2024c; Riddle & Di, 2020). Particularly, women’s lack of perception of the seriousness of IPV is one of the main reasons they stay in a relationship or tolerate violence (Fanslow & Robinson, 2010).

Likewise, attributed self-responsibility refers to an individual’s tendency to take responsibility for their actions, decisions, and the resulting consequences, whether positive or negative (Maß, 2022). In IPV, women could attribute more responsibility to themselves and, consequently, decide to stay in the violent relationship, making this variable an important role in their decision-making (Badenes-Sastre et al., 2024a, 2025).

According to Badenes-Sastre et al. (2025), both variables, perceived severity and attributed self-responsibility, could be considered cognitive distortions, that is, strategies used to reduce the discomfort the cognitive dissonance between the situation experienced (e.g., IPV) and beliefs (e.g., love can do all things; Aiquipa Tello & Canción Suárez, 2020; Festinger, 1957) causes. Cognitive distortions could significantly influence decision-making in violent relationships, causing women to reevaluate situations of violence, minimize IPV, and place responsibility for IPV on themselves (Badenes-Sastre et al., 2025). Given the critical role these cognitive distortions play in the experiences of women affected by IPV, identifying the factors that predict their occurrence is essential. A deeper understanding of these predictive variables could help elucidate the mechanisms underlying cognitive distortions and inform future interventions aimed at fostering adaptive decision-making processes among women in violent relationships.

## 3. Predictors of Perceived Severity and Attributed Self-Responsibility

Various factors may predict the emergence of cognitive distortions—specifically, the perception of severity and the attribution of self-responsibility—and can function as risk factors for women IPV victims. There is empirical evidence that such distortions may help minimize and justify violence and increase tolerance of it (Bonilla-Algovia & Rivas-Rivero, 2021; Guerrero-Molina et al., 2023), reinforcing the cycle of abuse and increasing the likelihood of victims remaining in the relationship, thereby endangering their safety, well-being, and lives. Conversely, certain factors may function as protective factors (Dim & Elabor-Idemudia, 2021) by enhancing the perception of the severity of violence and the acknowledgment of the attribution of self-responsibility. The main variables that may influence cognitive distortions are ideological, relational, and sociodemographic factors (Badenes-Sastre et al., 2024b; Bucheli & Rossi, 2017; Garrido-Macías et al., 2020; Gracia et al., 2009a, 2009b; Lelaurain et al., 2021; Tan et al., 2018; Vidal-Fernández & Megías, 2014). Ideological variables include ambivalent sexism, attitudes toward violence, and feminist identification. Relational variables involve dependency on the partner and commitment to the relationship. Lastly, sociodemographic factors are age, educational level, and prior experiences of IPV.

### 3.1. Ideological Variables

Regarding ideological variables, ambivalent sexism serves to justify and reinforce patriarchal norms and traditional gender roles (Glick & Fiske, 2012; Yang et al., 2023). It is widely recognized as a significant contributing factor to the occurrence of IPV (Garrido-Macías et al., 2017; Ibabe et al., 2016; Sánchez-Hernández et al., 2020). Likewise, ambivalent sexism is closely related to favorable attitudes toward IPV (Arnoso et al., 2022), which is another main barrier to the victim leaving the violent relationship (Arnoso et al., 2022; Lelaurain et al., 2017). Higher levels of adherence to ambivalent sexist beliefs and favorable attitudes toward IPV are associated with minimizing the perceived severity of IPV acts and attributing responsibility to the victim (Bucheli & Rossi, 2017; Lelaurain et al., 2021; Sánchez-Hernández et al., 2020; Valor-Segura et al., 2011; Vidal-Fernández & Megías, 2014), requiring consideration.

Furthermore, feminist identification may serve as a protective factor for women’s safety, well-being, and lives by providing a theoretical framework for understanding IPV through the gender dynamics and power relations in society (Dim & Elabor-Idemudia, 2021). This perspective illustrates how patriarchy contributes to the normalization and perpetuation of violence (Dim & Elabor-Idemudia, 2021). Feminist perspectives advocate for shifting the blame away from victims and holding aggressors responsible (Hegarty & Tarzia, 2019) while also raising awareness about violence and its consequences (Nikupeteri et al., 2022). Consequently, feminist identification may enhance the perception of IPV severity and reduce attribution of self-responsibility, thus becoming a protective factor for women.

### 3.2. Relational Variables

Relational variables have been extensively studied in the field of IPV. One of the most studied dependencies on the partner refers to an individual’s deep emotional reliance on their partner in a relationship, characterized by a continual need for support, reassurance, and companionship (Aiquipa, 2015; Badenes-Sastre et al., 2024a; Beltrán-Morillas et al., 2019; Garrido-Macías et al., 2020; Rusbult & Arriaga, 1997; Valor-Segura et al., 2014). High dependency scores were associated with low perceived severity of violence (Badenes-Sastre et al., 2024a). Additionally, women who exhibit high levels of dependency tend to assign a greater attribution of self-responsibility, often blaming themselves for the problems in their IPV relationships (Beltrán-Morillas et al., 2019; Valor-Segura et al., 2014). Similarly, elevated levels of dependency are often associated with greater commitment to the relationship (Tan et al., 2018), which may lead women to minimize the severity of violent events (Garrido-Macías et al., 2020), increasing the probability that they will maintain the violent relationship (Garrido-Macías et al., 2020; Tan et al., 2018). This commitment to the relationship, as with high dependency (Beltrán-Morillas et al., 2019), could contribute to an increase in the attributed self-responsibility regarding violent behaviors against women.

### 3.3. Sociodemographic Variables

In terms of sociodemographic variables, societal progress has led to greater access to information about IPV. Consequently, with higher levels of education, women today may be better equipped to perceive the severity of such violence (Gracia et al., 2009b). Even though societal awareness regarding IPV has grown, incidence rates in Spain continue to rise rather than decline. In 2023, cases increased by 12.1% compared to the previous year, with 47.8% of victims aged between 30 and 44 years (Instituto Nacional de Estadística [INE], 2024). Although research indicates that women aged 25 to 45 perceive IPV as more severe than do older individuals (Gracia et al., 2009a), this may be attributed to heightened awareness of more overt forms of violence, such as physical or sexual violence. However, they may be less likely to recognize more subtle and increasingly prevalent forms of IPV, such as controlling behaviors facilitated by technology (Sánchez-Hernández et al., 2020).

There is empirical evidence to suggest that women with experiences of IPV (that is, women who have previously lived through situations of violence) tend to perceive violence experienced as more severe than those who have not experienced IPV (Badenes-Sastre et al., 2024c; Fiorillo-Ponte, 1999). Additionally, women who have been more exposed to violent behavior report higher levels of attributed self-responsibility (Reich et al., 2015). Furthermore, having witnessed their mother suffer IPV makes women more likely to experience IPV situations when they are adults (Ludermir et al., 2017). The experience of IPV situations can influence perceived severity and attributed self-responsibility, making women more lenient toward acts of violence they experience, justifying and reinterpreting the aggressor’s behaviors to maintain the relationship (Goodfriend & Arriaga, 2018).

### 3.4. Role of Cognitive Distortions Based on Type of IPV

Different forms of IPV are perceived with different degrees of severity. The traditional conceptualization of IPV as primarily physical or sexual may contribute to the perception of these forms of violence as more severe than psychological violence or controlling behaviors (Lelaurain et al., 2018; Walker et al., 2021; Yamawaki et al., 2018). Moreover, the severity of sexual violence is often perceived differently depending on the perpetrator. For instance, sexual violence committed by a stranger tends to be regarded as more severe than that perpetrated by a partner (Diakonova-Curtis, 2013; Gomez-Pulido et al., 2024).

Likewise, the responsibility attributed to violence may vary depending on the typology. Findings suggest that perpetrators of physical violence are typically attributed greater responsibility than those involved in sexual violence, psychological violence, or controlling behaviors (Badenes-Sastre et al., 2023a). However, there is limited research addressing attributed self-responsibility among victims, particularly concerning how it varies across types of IPV. Therefore, we aim to further explore the perceived severity and attributed self-responsibility and thereby contribute to a deeper understanding of these variables that are under-researched.

## 4. The Present Study

The purpose of this study was to analyze which variables predict the perceived severity of IPV and attributed self-responsibility of women according to IPV manifestation (physical, psychological, sexual violence, and controlling behaviors). Specifically, we analyzed the predictor role of ideological (attitudes toward IPV, ambivalent sexism, and feminist identification), relational (dependency and commitment to the relationship), and sociodemographic (educational level, age, and previous experiences of IPV) variables in cognitive distortions (perceived severity and attributed self-responsibility).

As an additional objective, we examined differences among violence types (physical, psychological, sexual violence, and controlling behaviors) in perceived severity and attributed self-responsibility. Lastly, auxiliary analyses were conducted to examine potential differences in these variables between women who had experienced IPV within the past 12 months and those who had encountered it at some point in their lifetime.

## 5. Method

### 5.1. Design and Procedure

An exploratory study was conducted with an intra-subject design. An online survey was designed using the Qualtrics platform and disseminated in Spain through social networks (WhatsApp, Instagram, and Facebook) and institutional email [University of Granada]. Participants were selected through incidental sampling. The inclusion criteria for this study were as follows: participants had to be women aged 18 or older, of Spanish nationality, and currently in or having previously been in a relationship with a man.

To classify participants as victims or non-victims, they first completed the WHO Violence Against Women Instrument. Subsequently, they responded to four situations based on items from this instrument, each focusing on a specific type of violence: physical violence, sexual violence, psychological violence, and controlling behavior. They were asked about the degree of perceived severity, attributed self-responsibility, and familiarity with each of the IPV types. Then, they completed the other measures of interest. Participants were informed about the study’s objectives. They provided informed consent in accordance with the Declaration of Helsinki, ensuring anonymity and confidentiality. Participants did not receive any monetary or other incentives for their participation. The study was conducted with the approval of the Ethics Committee of the University of [University of Granada].

### 5.2. Participants

The sample size was determined a priori using the software G*power (Faul et al., 2009). For a linear multiple regression analysis with a fixed model and R^2^ increase, assuming a medium effect size of 0.15 (Bologna, 2022), a significance level of α = 0.05 (two-tailed), and a desired statistical power of 0.80 (Cárdenas & Arancibia, 2014), the calculation indicated that minimum of 77 participants was necessary. The initial sample consisted of 373 women, of whom 116 were excluded due to incomplete surveys or being under 18 years old. Consequently, the final sample comprised 257 women (*M* = 28.61, *SD* = 9.79; age range 18–75), of whom 191 stated they had suffered physical violence, sexual violence, psychological violence, or controlling behaviors, as measured by the WHO’s Violence Against Women Instrument (vs. 66 no informed IPV). Finally, most participants had a university education (89.5% vs. 10.5% non-university education).

### 5.3. Instruments

**IPV Against Women.** To assess whether they had been victims of IPV, the WHO’s Violence Against Women Instrument (Badenes-Sastre et al., 2023b) (see Supplementary Materials) was applied. This instrument includes 25 items that assess experiences of violent behaviors perpetrated by a male partner or ex-partner. It consists of four subscales, each measuring a different form of violence: nine items assess physical violence (e.g., “Has your partner or former partner slapped you?”), seven assess psychological violence (e.g., “Has your partner or former partner insulted you?”), three assess sexual violence (e.g., “Has your partner or former partner forced you to have sexual intercourse when you did not want to?”), and six assess controlling behaviors (e.g., Has your partner or former partner tried to restrict contact with your family of birth?”). In this study, participants were told to imagine a situation of IPV performed by their male partner or ex-partner. This request was repeated four times, once for each type of violence—physical, psychological, sexual, and controlling behaviors. For example, one request was, **“Imagine that your partner engages in any of these behaviors with you [physical, psychological, sexual or control items of WHO violence against women instrument]”**. Participants responded to questions about perceived severity, attributed self-responsibility, and the experience of someone close to them.

**Cognitive Distortions.** To assess cognitive distortions, perceived severity, and attributed self-responsibility, the following measures were employed:

- Perceived Severity. Based on the previous literature (Badenes-Sastre et al., 2023a, 2023b; Garrido-Macías et al., 2017; Sánchez-Hernández et al., 2020), perceived severity was measured by asking, “To what extent do you perceive the behavior as serious?” Participants responded on a 7-point response scale (1 = nothing severe, 7 = very severe). This question was posed four times, corresponding to each type of violence evaluated (physical, sexual, psychological violence, and controlling behaviors).
- Attributed Self-responsibility. Drawing on the previous literature (Badenes-Sastre et al., 2024b; McCullough et al., 2003) that assessed responsibility attributed to the aggressor, the measurement approach was adapted to align with the objectives of the present study, focusing on the assessment of attributed self-responsibility. Participants were asked, “To what extent do you consider yourself responsible for these behaviors?” They responded on a scale from 1 (not at all responsible) to 7 (very responsible), applying this evaluation individually for each violence type: physical, sexual, psychological, and controlling behaviors.

**Experiences of IPV (Someone Close).** An item was developed to assess experience indirectly through exposure to another person’s experiences. Specifically, a single-item measure was used to evaluate participants’ familiarity with violent behavior by close contact: “To what extent have people in your close environment (mother, sister, friends, etc.) experienced the behaviors presented?” Participants were asked to answer “yes” or “no”. This item was administered separately for each of the four types of IPV examined.

**Favorable Attitudes Toward IPV**. To evaluate the degree of favorable attitudes toward IPV among participants, the Intimate Partner Violence Attitude Scale Revised (Pastor et al., 2020) was utilized, which consists of 17 items with a 7-point Likert-type response scale (1 = Strongly disagree, 5 = Strongly agree) to assess favorable attitudes toward IPV, categorized into three subscales. The scale includes eight items related to attitudes toward abuse (e.g., “It is okay for me if I blame my partner when I do things wrong”), four items pertaining to attitudes about violence (e.g., “It would never be appropriate to hit or try to hit your partner with an object”), and five items about attitudes toward controlling behavior (e.g., “I would be flattered if my partner told me not to talk to someone of the opposite sex”). In the present study, the scale demonstrated a Cronbach’s alpha of 0.74.

**Feminist Identification.** To examine the extent of feminist identification, the Feminist Identification Scale (De Lemus et al., 2015) was administered. It comprises four items (e.g., “I identify with the Feminist”). Participants rated their agreement on a 7-point Likert scale (1 = Totally disagree, 7 = Totally agree). Cronbach’s alpha was 0.95.

**Ambivalent Sexism.** The Ambivalent Sexism Inventory (Expósito et al., 1998) was employed to evaluate the level of ambivalent sexism. It consists of 22 items rated on a 6-point Likert-type scale (0 = Totally disagree, 5 = Totally agree). It is divided into two subscales: benevolent sexism (e.g., “In the event of a catastrophe, women should be rescued before men”) and hostile sexism (e.g., “Feminist women are making completely unreasonable demands on men”). Cronbach’s alpha in our sample was 0.92.

**Dependency on the Partner.** To evaluate dependency on the partner, the Spouse-Specific Dependency Scale (Valor-Segura et al., 2009) was applied. This scale consists of 17 items to evaluate partner-specific dependency across three dimensions: six items concerning emotional dependency (e.g., “Having a relationship with my partner makes me feel safe”), five items related to anxious attachment (e.g., “I feel bad if my partner has a good time without me”), and six items concerning exclusive dependency (e.g., “My partner is the only one I could turn to in case of a problem”). Participants responded on a 7-point Likert scale (1 = Totally disagree, 7 = Totally agree). Cronbach’s alpha for our sample was 0.82.

**Commitment to the Relationship.** We selected the three items corresponding to commitment to the relationship (e.g., “How faithful are you in your relationship?”) from the Perceived Relationship Quality Components Scale (Fletcher et al., 2000). Participants rated their responses on a Likert-type scale (1 = Not at all, 7 = Very much). Cronbach’s alpha was 0.84.

**Sociodemographic Variables.** Participants were asked to provide their age and education level. For age, they were asked to indicate their age by entering a number corresponding to their current age. Regarding educational level, they were asked to indicate whether they had completed university studies or not.

### 5.4. Analysis Strategy and Data Extraction

The data were analyzed using the Statistical Package for the Social Sciences program, version 28. Initially, frequency analysis was conducted to examine the prevalence of IPV throughout one’s lifetime and in the past 12 months in our sample. Then, a stepwise linear regression was conducted to determine which variables best predicted the perceived severity and attributed self-responsibility among women based on the IPV type (physical, sexual, psychological, and controlling behaviors). In the first step, ideological variables were added, such as favorable attitudes toward IPV, ambivalent sexism, and feminist identification. In the second step, relational variables such as dependency with partner and commitment to the relationship were added. Finally, in the third step, sociodemographic variables were included, such as being a victim (or not), familiarity with violent behavior (experiences of IPV by someone close), and age and educational level. Finally, a repeated-measures linear model was performed to explore the possible differences in the perceived severity of IPV and attributed self-responsibility based on the IPV type.

## 6. Results

### 6.1. Prevalence of IPV

Of the 257 women surveyed, 191 (74.3%) stated they had experienced some form of IPV by their current or ex-partner. Specifically, 24.5% of women claimed to have experienced physical violence, 32.3% sexual violence, 59.5% psychological violence, and 62.3% controlling behaviors by their partner or ex-partner at some time in their life. Among women who had experienced IPV, 53.9% had done so in the past 12 months (7.9% had suffered physical violence, followed by 14.1% sexual violence, 40.8% psychological violence, and 38.7% controlling behaviors).

### 6.2. Predictors of Perceived Severity of IPV and Attributed Self-Responsibility

Stepwise linear regression was applied to test the predictors of perceived severity (Table 1) and attributed self-responsibility (Table 2) per IPV type (physical, sexual, and psychological violence and controlling behaviors).

**Physical violence.** The model explaining perceived severity identified significant predictors across various variables, specifically favorable attitudes toward IPV (β = −0.33, *p* = 0.01), ambivalent sexism (β = −0.40, *p* < 0.001), feminist identification (β = 0.06, *p* = 0.01), commitment (β = 0.13, *p* < 0.001), and educational level (β = 0.19, *p* = 0.01). In the model predicting attributed self-responsibility, ideological, relational, and sociodemographic variables were not significant predictors.

**Sexual violence.** The model demonstrated that multiple variables influenced perceived severity, namely favorable attitudes toward IPV (β = −0.51, *p* < 0.001), commitment to the relationship (β = 0.09, *p* = 0.00), and age (β = 0.00, *p* = 0.00). For the criterion variable of attributed self-responsibility, being a victim was the only significant predictor (β = 0.58, *p* = 0.00).

**Psychological violence.** In the predictive model for perceived severity, the results indicated favorable attitudes toward IPV (β = −0.44, *p* = 0.00) and dependency (β = 0.20, *p* = 0.00). The predictive model for attributed self-responsibility revealed that commitment (β = −0.19, *p* = 0.02), dependency (β = 0.30, *p* = 0.02), being a victim (β = 0.50, *p* = 0.01), and age (β = −0.02, *p* = 0.02) significantly contributed to attributed self-responsibility.

**Controlling behaviors.** The predictive approach for perceived severity indicated that favorable attitudes toward IPV (β = −0.64, *p* < 0.001), feminist identification (β = 0.05, *p* = 0.02), and commitment (β = 0.09, *p* = 0.04) were identified as significant predictors. The model for attributed self-responsibility demonstrated that ambivalent sexism (β = 0.40, *p* < 0.001) and favorable attitudes toward IPV (β = 0.72, *p* = 0.03) were significant predictors.

### 6.3. Differences in Perceived Severity and Attributed Self-Responsibility According to Type of IPV

A one-way repeated measures analysis of variance was conducted to compare the perceived severity and attributed self-responsibility by women in each IPV type (physical, sexual, psychological violence, and controlling behaviors). Differences were found in both variables, perceived severity and attributed self-responsibility (see Table 3). Specifically (see Table 4), physical violence was perceived as more severe, *M* = 6.91, Wilks’ lambda = 0.86, *F* (3, 254) = 13.92, *p* < 0.001, *η*^2^*p* = 0.14, than psychological violence (*M* = 6.75), and controlling behaviors (*M* = 6.62). However, it did not differ significantly from sexual violence (*M* = 6.84). Likewise, significant differences were found in attributed self-responsibility for IPV. Specifically, controlling behaviors was the type of violence for which participants most frequently attributed self-responsibility, *M* = 1.97, Wilks’ lambda = 0.87, *F* (3, 254) = 12.814, *p* < 0.001, *η*^2^*p* = 0.13, compared to physical violence (*M* = 1.48), sexual violence (*M* = 1.80), and psychological violence (*M* = 1.91).

### 6.4. Auxiliary Analyses: Differences in Perceived Severity and Attributed Self-Responsibility According to Recent vs. Lifetime IPV Experiences

An independent sample *t*-test was conducted to examine the differences in perceived severity and attributed self-responsibility by type of violence, based on when women experienced these types of violence (within the last 12 months vs. throughout their lifetime; Table 5).

Regarding the perceived severity of the types of violence, we found significant differences only in sexual violence. Women who experienced sexual violence in the last 12 months perceived it as less severe than those who had experienced it throughout their lifetime (*M* = 6.78 vs. *M* = 6.90, *p* = 0.03). No significant differences were found in terms of attributed self-responsibility.

## 7. Discussion

In the present study, we explored which ideological, relational, and sociodemographic variables most strongly predicted perceived severity and attributed self-responsibility of IPV in women, differentiating by type of violence (physical, sexual, psychological violence, and controlling behaviors). The main findings indicated that ambivalent sexism, attitudes toward IPV, feminist identification, dependency, commitment to the relationship, educational level, age, and being a victim of IPV in general were significant predictors of perceived severity or attributed self-responsibility in some types of IPV.

Regarding the cognitive distortion of perception of the severity of IPV, ambivalent sexism was a significant predictor of perceived severity in cases of sexual violence. This is consistent with the previous literature indicating that ambivalent sexism contributes to the minimization of the perceptions of the severity of IPV in general (Lelaurain et al., 2021). Additionally, favorable attitudes toward IPV consistently predicted lower perceived severity across all types of violence (Lelaurain et al., 2021; León & Aizpurua, 2023). Finally, feminist identification emerged as a predictor in cases of physical violence and controlling behaviors. This finding aligns with the literature suggesting that feminist ideology facilitates the recognition of violence (Dim & Elabor-Idemudia, 2021), thereby influencing the perception of its severity. However, further investigation is necessary to clarify these findings.

Regarding relational variables, dependency predicted perception of severity in cases of psychological violence and commitment in cases of physical violence, sexual violence, and controlling behaviors. According to previous research (Badenes-Sastre et al., 2024a; Gilbert & Gordon, 2017), women victims who exhibit higher levels of dependency and commitment are more likely to minimize or perceive IPV as less severe. However, contrary to expectations and the existing literature, the current findings indicate that women with lower dependency (in cases of psychological violence) and lower commitment (in cases of physical, sexual, and controlling behaviors) perceived less severity of IPV, minimizing it and thus increasing cognitive distortion. These results suggest the need for further investigation to clarify these unexpected outcomes, for they may indicate that these variables are not relevant for predicting perceived severity but are significant for other factors, such as decision-making processes.

Lastly, the sociodemographic variables that predicted perceived severity were educational level for physical violence and age for sexual violence. These findings align with the existing literature, which suggests that women with higher educational attainment tend to perceive violence as more severe (Gracia et al., 2009a), decreasing cognitive distortion. Additionally, age emerged as a significant predictor in cases of sexual violence, with younger women perceiving lower severity. This result supports previous research indicating that younger individuals are more likely to perceive IPV as less severe, possibly due to a greater inclination toward favorable attitudes toward IPV (Martín-Fernández et al., 2022). In younger populations, this tendency may also be attributed to the normalization of sexual violence through pornography consumption (Gallego Rodríguez & Fernández-González, 2019). Moreover, social media plays a crucial role by exposing users to traditional gender stereotypes and rape myths, which perpetuate sexual violence (Sarmiento, 2023).

With respect to the cognitive distortion of attributed self-responsibility, ambivalent sexism and attitudes toward IPV were significantly associated with increased attributed self-responsibility in control behavior. According to the literature (Badenes-Sastre et al., 2023a; Cinquegrana et al., 2022; Lelaurain et al., 2018), women victims of IPV with higher levels of ambivalent sexism and more permissive attitudes toward IPV tend to assign greater responsibility to the victim of IPV, for these ideologies contribute to the normalization and acceptance of violence, often leading to the perception that the victim is more responsible than the perpetrator.

Among the relational variables, dependency and commitment emerged as predictors in the context of psychological violence. With respect to dependency and in line with the previous literature, women with higher dependency tended to indicate greater attributed self-responsibility in their IPV experiences (Beltrán-Morillas et al., 2019; Valor-Segura et al., 2014). Furthermore, with respect to the commitment, one might logically expect that higher relationship commitment—reflecting a greater investment of resources (Rusbult & Martz, 1995)—would lead individuals to assume more attributed self-responsibility in maintaining the relationship. However, the results indicate that women with lower commitment in the face of psychological violence exhibited higher levels of attributed self-responsibility. These novel findings suggest a need for further investigation into how commitment influences attributed self-responsibility in the current cultural context, particularly given that women’s perceptions of their IPV experiences are shaped by various sociocultural and personal factors (Vass et al., 2024).

Regarding sociodemographic variables, the predictor of attributed self-responsibility was “being a victim” in cases of sexual and psychological violence. Specifically, the findings indicated that women who had been victims of IPV were more likely to take responsibility for it. This observation is consistent with previous research, which suggests that increased exposure to violent behavior is associated with higher levels of attributed self-responsibility (Reich et al., 2015). On the other hand, the existing literature indicates that older women tend to attribute more responsibility to victims, a phenomenon linked to the persistence of rigid gender stereotypes and cultural myths (Tang et al., 2002). However, our findings suggest that in the context of psychological violence, younger women were more inclined to attribute self-responsibility to IPV. This result aligns with recent research indicating that younger individuals are more prone to attribute self-responsibility, potentially due to a greater propensity to justify violence and more pronounced sexist attitudes (Sánchez-Prada et al., 2020; Waltermaurer, 2012; Wang, 2016) and the knowledge that young people can have through the use of social media (Sarmiento, 2023). Given the contradictory findings in the literature, the identification of age as a predictor in attributed self-responsibility is a key contribution of this study, offering clarity and new insights into the field.

The results regarding the attributed self-responsibility for physical violence did not show the presence of statistically significant predictors. This lack of significance may arise because its overt nature makes it more easily identifiable, leaving physical and visible evidence (Delker et al., 2022), leading to less ambiguity in the attributed self-responsibility compared to more subtle forms of violence, in which the attributed self-responsibility may vary more due to the difficulty of recognizing such behaviors (Novo et al., 2016).

As an additional objective, we explored which types of IPV were perceived as more severe and to which women attributed more self-responsibility. Specifically, physical violence had the highest perceived severity in our study, which again reflects that physical or sexual violence is usually perceived as more severe than more subtle violence, such as psychological and controlling behaviors (Medinilla-Tena et al., 2024; Novo et al., 2016; Walker et al., 2021). It is consistent with the fact that subtle violence is not considered to the same extent as more overt violence, making it less likely to be detected and its severity not perceived or minimized (Buesa & Calvete, 2011; Novo et al., 2016). Perceiving these forms of violence as less severe can be dangerous because a distorted interpretation of reality can lead to a failure to recognize the risk to personal integrity (Badenes-Sastre & Expósito, 2021). Otherwise, it was found that controlling behaviors were the form of violence in which women attributed the most responsibility to themselves, compared to physical, sexual, and psychological violence. This can be explained in the sense that controlling behaviors constitute subtle forms of violence, which make identification difficult (Medinilla-Tena et al., 2024). It should be noted that the new modalities of socialization mediated by technology, especially those manifested through phones, have helped normalize and tolerate these behaviors (Sánchez-Hernández et al., 2020). This can lead women to assume more responsibility, given that their partner can continuously and unrestrictedly access their personal data, violating their privacy (Sánchez-Hernández et al., 2024). Consequently, women may feel that they are responsible for allowing for these behaviors, believing that they are romantic acts (Sánchez-Hernández et al., 2020).

Lastly, we examined differences in perceived severity and attributed self-responsibility among women who had experienced IPV in their lifetime or in the past 12 months. We found that only sexual violence was perceived as less severe among women who had experienced IPV in the past 12 months. It seems that previous studies have shown that women who were involved in a current relationship showed increased tolerance for violent sexual behavior (Garrido-Macías et al., 2022). In addition, women who experience sexual IPV perpetrated by a person with whom they have a close relationship tend to reinterpret the behaviors by minimizing and justifying them (Garrido-Macías et al., 2022).

### Limitations and Future Directions

Although this study presents novel findings, it is not without limitations. One of the main limitations is related to the sample, which is comprised of young people, most of whom are university-educated. This demographic characteristic could influence the variables studied. In addition, the current design did not allow for an in-depth examination of psychological and controlling behaviors despite their particularly high prevalence among young people (Fernández-Ramos, 2020). Because these forms of violence often manifest subtly, participants may under-recognize their severity and even come to view themselves simultaneously as both victim and responsible party (Medinilla-Tena et al., 2024). Future work should, therefore, prioritize a more detailed investigation of psychological and controlling behaviors to determine how their subtle nature influences perceptions of severity and attribution of self-responsibility.

Another limitation is that the survey did not randomly present the items corresponding to each type of IPV, so all participants saw them in the same order, which may have influenced the results obtained. Moreover, women who stated they had experienced IPV in the past 12 months claimed to have experienced IPV earlier in their lives, reflecting a repetitive pattern. Previous studies estimated that between 40% and 60% of women who were victims of IPV experienced new assaults by their partner or ex-partner, especially if the incident occurred within the first 6 months of the initial violent behavior (Muñoz-Rivas et al., 2024; Tomkins et al., 2023). In future research, it would be interesting to examine the maintenance of cognitive distortions, taking into account temporality, whether they were experienced only in the past or more recently.

Researchers should consider examining the collective influence of all variables analyzed in this study on IPV as a whole rather than studying specific types of violence. IPV is a multidimensional phenomenon that often co-occurs across various forms, making its separation for predictive purposes potentially counterproductive. This fragmentation may contribute to the variability and contradictions observed in the results. Therefore, adopting a comprehensive approach could enhance the identification of key predictors of perceived severity and attributed self-responsibility, providing a more accurate understanding of the cognitive distortions associated with IPV. It would also be relevant to consider additional factors, such as a deeper knowledge of feminist ideology and explaining its meaning beforehand, because, in the present study, participants were only asked whether they identified with it. Today, feminist ideology seems to be interpreted differently, which could lead each person to associate the concept with their own interpretation, thus affecting the results obtained.

Future studies could also consider other factors related to health problems or mental disorders that may result from IPV, such as depression, anxiety, eating disorders, and alcohol consumption (World Health Organization, 2024), as these may influence the cognitive distortions developed by women in violent situations.

In conclusion, it would be of great interest to develop specific intervention programs aimed at recognizing and addressing the cognitive distortions that frequently arise in IPV situations. These programs could be designed with a dual approach: (a) to equip women survivors of IPV with skills to recognize and understand these distortions, thereby enhancing their adaptive responses, and (b) to provide preventive strategies to women without IPV experience to develop resilience and identify dysfunctional thinking patterns in relationships. This preventive approach could contribute to greater awareness and understanding of the effects of cognitive distortions and their role in power and control dynamics in IPV.

## 8. Conclusions

The perceived severity of IPV and the attributed self-responsibility are two crucial factors for women victims in IPV situations because they can lead women to normalize and minimize the situation of violence and keep them in the violent relationship. At this point, this study’s results indicate that sociodemographic, ideological, and relational variables significantly predict the perception of severity and attributed self-responsibility in the context of IPV. These findings provide valuable information about the complexity of the situations women victims face and highlight the need for further research on these factors because they constitute critical barriers to ending violent relationships. Despite advances in raising awareness of IPV, the results suggest that although individuals may recognize the severity of more traditional forms of violence, such as physical aggression, other forms, such as controlling behaviors, tend to be normalized. This indicates that although societal awareness has progressed, cognitive distortions continue to play a role in justifying and minimizing IPV, ultimately hindering victims’ recognition of abuse and their ability to exit violent relationships.

## Figures and Tables

**Table 1:** Predictors of Perceived Severity According to the Type of Violence.

	Perceived Severity			
	Physical violence			
Predictors	R^2^	*b*	*t*	95% CI
*Step 1*	0.02 *			
Attitudes towards IPV		−0.33	−2.38	[−0.61, −0.05]
*Step 2*	0.03 **			
Attitudes towards IPV		−0.40	−2.83	[−0.68, −0.12]
Ambivalent sexism		0.12	2.05	[0.00, 0.24]
*Step 3*	0.06 **			
Attitudes towards IPV		−0.37	−2.66	[−0.65, −0.09]
Ambivalent sexism		0.07	3.12	[0.08, 0.36]
Feminist identification		0.02	2.53	[0.01, 0.12]
*Step 4*	0.10 ***			
Attitudes towards IPV		−0.29	−2.06	[−0.57, −0.13]
Ambivalent sexism		0.20	2.90	[0.06, 0.34]
Feminist identification		0.07	2.71	[0.01, 0.12]
Commitment to the relationship		0.13	3.43	[0.05, 0.20]
*Step 5*	0.12 ***			
Attitudes towards IPV		−0.23	−1.67	[−0.51, 0.04]
Ambivalent sexism		0.21	3.01	[0.07, 0.35]
Feminist identification		0.06	2.62	[0.01, 0.11]
Commitment to the relationship		0.12	3.31	[0.05, 0.19]
Educational level		0.19	2.54	[0.04, 0.33]
	Sexual violence			
Predictors	R^2^	*b*	*t*	95% CI
*Step 1*	0.08 ***			
Attitudes towards IPV		−0.51	−4.91	[−0.72, −0.31]
*Step 2*	0.12 ***			
Attitudes towards IPV		−0.46	−4.46	[−0.67, −0.26]
Commitment to the relationship		0.09	3.19	[0.03, 0.14]
*Step 3*	0.14 ***			
Attitudes towards IPV		−0.49	−4.78	[−0.70, −0.29]
Commitment to the relationship		0.09	3.23	[0.03, 0.14]
Age		0.00	2.69	[0.00, 0.01]
	Psychological violence			
Predictors	R^2^	*b*	*t*	95% CI
*Step 1*	0.03 **			
Attitudes towards IPV		−0.44	−2.83	[−0.75, −0.13]
*Step 2*	0.06 ***			
Attitudes towards IPV		−0.57	−3.54	[−0.89, −0.25]
Dependency		0.20	2.81	[0.06, 0.34]
	Control behaviors			
Predictors	R^2^	*b*	*t*	95% CI
*Step 1*	0.06 ***			
Attitudes towards IPV		−0.64	−4.23	[−0.94, −0.34]
*Step 2*	0.08 ***			
Attitudes towards IPV		−0.57	−3.73	[−0.87, −0.27]
Feminist identification		0.05	2.23	[0.00, 0.10]
*Step 3*	0.10 ***			
Attitudes towards IPV		−0.51	−3.32	[−0.82, −0.21]
Feminist identification		0.06	2.47	[0.01, 0.10]
Commitment to the relationship		0.09	2.33	[0.01, 0.18]

*Note.* IPV = intimate partner violence; N = 257; * *p* < 0.05, ** *p* < 0.01, *** *p* < 0.001. Only statistically significant variables are reported by the model; non-significant variables are omitted from the results output.

**Table 2:** Predictors of Self-Responsibility Attributed According to the Type of Violence.

	Self-Responsibility Attributed			
	Sexual violence			
Predictors	R^2^	*b*	*t*	95% CI
*Step 1*	0.02 **			
Being a victim (or not)		0.58	2.68	[0.15, 1.02]
	Psychological violence			
Predictors	R^2^	*b*	*t*	95% CI
*Step 1*	0.02 *			
Commitment to the relationship		−0.19	−2.31	[−0.36, −0.02]
*Step 2*	0.03 **			
Commitment to the relationship		−0.23	−2.74	[−0.40, −0.06]
Dependency		0.30	2.20	[0.03, 0.58]
*Step 3*	0.06 **			
Commitment to the relationship		−0.20	−2.40	[−0.37, −0.03]
Dependency		0.27	1.98	[0.00, 0.55]
Being a victim (or not)		0.50	2.47	[0.10, 0.91]
*Step 4*	0.08 ***			
Commitment to the relationship		−0.20	−2.43	[−0.37, −0.03]
Dependency		0.25	1.86	[−0.01, 0.53]
Being a victim (or not)		0.49	2.44	[0.09, 0.90]
Age		−0.02	−2.29	[−0.03, −0.00]
	Control Behaviors			
Predictors	R^2^	*b*	*t*	95% CI
*Step 1*	0.02 **			
Ambivalent sexism		0.40	2.77	[0.11, 0.69]
*Step 2*	0.04 **			
Ambivalent sexism		0.32	2.20	[0.03, 0.62]
Attitudes towards IPV		0.72	2.09	[0.04, 1.41]

*Note.* IPV = intimate partner violence; N = 257; * *p* < 0.05, ** *p* < 0.01, *** *p* < 0.001. Only statistically significant variables are reported by the model; non-significant variables are omitted from the results output.

**Table 3:** Repeated Measure Comparing Perceived Severity and Attributed Self-Responsibility According to The Type of Violence.

	Physical Violence		Sexual Violence		Psychological Violence		Control Behaviors					
	*M*	*SD*	*M*	*SD*	*M*	*SD*	*M*	*SD*	*df*	*F*	*p*	*Partial eta Squared*
Perceived severity	6.91	0.67	6.84	0.52	6.75	0.75	6.62	0.74	3.254	13.92	<0.001	0.14
Attributed self-responsibility	1.48	1.30	1.80	1.56	1.91	1.46	1.97	1.63	3.254	12.81	<0.001	0.13

*Note.* N = 257.

**Table 4:** Pairwise Comparisons by Types of Violence in Perceived Severity and Self-Responsibility Attributed.

Perceived Severity				
Comparison	Mean difference	SE	*p*	95% CI
Physical violence vs. Sexual violence	0.07	0.04	0.53	[−0.03, 0.17]
Physical violence vs. Psychological violence	0.15 *	0.05	0.01	[0.02, 0.29]
Physical violence vs. Control behavior	0.28 *	0.05	<0.001	[0.14, 0.42]
Sexual violence vs. Psychological violence	0.08	0.04	0.24	[−0.02, 0.19]
Sexual violence vs. Control behavior	0.21 *	0.03	<0.001	[0.12, 0.31]
Psychological violence vs. control behavior	0.13 *	0.04	0.03	[−0.25, −0.00]
Attributed Self-Responsibility				
Comparison	Mean difference	SE	*p*	95% CI
Physical violence vs. Sexual violence	−0.31 *	0.08	0.00	[−0.55, −0.08]
Physical violence vs. Psychological violence	−0.43 *	0.08	<0.001	[−0.64, −0.22]
Physical violence vs. Control behavior	−0.48 *	0.09	<0.001	[−0.73, −0.23]
Sexual violence vs. Psychological violence	−0.11	0.09	1.00	[−0.36, −0.13]
Sexual violence vs. Control behavior	−0.16	0.09	0.47	[−0.42, −0.08]
Psychological violence vs. control behavior	−0.05	0.08	1.00	[−0.29, 0.18]

*Note.* N = 257. * It corresponds to the data pairs that have significant mean differences.

**Table 5:** Differences in perceived severity and attributed self-responsibility among women who have had IPV experience (in the past 12 months vs. lifetime).

	Lifetime *n* = 88		In the Past 12 Months *n* = 103		Mean Difference	95% CI	*df*	*t*	*p*	Partial eta Squared
Perceived severity										
	*M*	*SD*	*M*	*SD*						
Physical violence	6.93	0.64	6.95	0.25	−0.02	[−0.15, 0.11]	189	−0.28	0.77	0.47
Sexual violence	6.90	0.30	6.78	0.46	0.12	[0.00, 0.23]	178.01	2.16	0.03	0.39
Psychological violence	6.74	0.70	6.76	0.53	−0.01	[−0.19, 0.15]	189	−0.20	0.83	0.61
Control behaviors	6.61	0.74	6.58	0.65	0.03	[−0.16, 0.23]	189	0.30	0.75	0.69
Self-Responsibility attributed										
	*M*	*SD*	*M*	*SD*						
Physical violence	1.40	1.00	1.64	1.50	−0.24	[−0.62, 0.13]	184.62	−1.25	0.20	1.33
Sexual violence	1.88	1.66	2.02	1.69	−0.14	[−0.62, 0.33]	189	−0.59	0.55	1.68
Psychological violence	1.90	1.50	2.21	1.53	−0.31	[−0.75, 0.12]	189	−1.43	0.15	1.52
Control behaviors	1.90	1.45	2.17	1.74	−0.26	[−0.73, 0.19]	189	−1.13	0.25	1.61

## Data Availability

The datasets generated during and/or analyzed during the current study are available from the corresponding author upon reasonable request.

## Supplementary Materials

The following supporting information can be downloaded at: https://www.mdpi.com/article/10.3390/bs15050677/s1.
