# Supplementary material for: Predictors of Cognitive Distortions in Intimate Partner Violence: An Analysis of Ideological, Relational and Sociodemographic Factors
Source: Behav Sci (Basel). 2025 May 15;15(5):677. doi: 10.3390/bs15050677 (PMC12109419; doi:10.3390/bs15050677)
Supplement: Supplementary file 1 [file behavsci-15-00677-s001.zip › behavsci-3577317-supplementary.pdf]

Supplementary Material

Spanish Version of the WHO Violence Against Women Instrument

Instrumento de la Organización Mundial de la Salud sobre la Violencia Contra las Mujeres

[World Health Organization Instrument on Violence Against Women]

A continuación, se le presentan una sucesión de preguntas que hacen referencia a experiencias o situaciones que pueden haber acontecido en su relación de pareja actual (si la tiene) o en una relación pasada con un hombre. Por favor, léalas con atención e intente responder de manera honesta a cada una de las cuestiones.

[Below are a series of questions that refer to experiences or situations that may have occurred in your current relationship (if you have one) or in a past relationship with a man. Please read them carefully and try to answer each question honestly].

1. Nunca 2. Una vez 3. Pocas veces 4. Muchas veces

[1. Never 2. One 3. Few 4. Many]

Alguna vez su pareja actual y/o pasada... [Has your current and/or past partner ever...]	A lo largo de la vida [Throughout life]				En los últimos 12 meses [In the past 12 months]	
1. ¿Le ha insultado? [1. Insulted you?]	1	2	3	4	Sí [Yes]	No [No]
2. ¿Le ha menospreciado delante de otras personas? [2. Belittled you in front of other people?]	1	2	3	4	Sí [Yes]	No [No]

3. ¿Le ha humillado delante de otras personas? [3. Humiliated you in fornt of other people?]	1	2	3	4	Sí [Yes]	No [No]
4. ¿Le ha hecho cosas para asustarle a propósito? [4. Did things to scare you on purpose?]	1	2	3	4	Sí [Yes]	No [No]
5. ¿Le ha hecho cosas para intimidarle a propósito? [5. Did things to intimidate you on purpose?]	1	2	3	4	Sí [Yes]	No [No]
6. ¿Le ha amenazado con hacerle daño? [6. Threatened to hurt you?]	1	2	3	4	Sí [Yes]	No [No]
7. Le ha amenazado con hacerle daño a alguien que le importa? [7. Threatened to hurt someone you care about?]	1	2	3	4	Sí [Yes]	No [No]
8. ¿Le ha abofeteado? [8. Slapped you?]	1	2	3	4	Sí [Yes]	No [No]
9. ¿Le ha arrojado algo con lo que podría lastimarle? [9. Thrown something at you that could hurt you?]	1	2	3	4	Sí [Yes]	No [No]
10. ¿Le ha empujado? [10. Pushed you?]	1	2	3	4	Sí [Yes]	No [No]
11. ¿Le ha golpeado con el puño? [11. Hit you with his fist?]	1	2	3	4	Sí [Yes]	No [No]

12. ¿Le ha golpeado con algo que pueda herirle o hacerle daño? [12. Hit you with something else that could hurt you?]	1	2	3	4	Sí [Yes]	No [No]
13. ¿Le ha arrastrado? [13. Dragged you?]	1	2	3	4	Sí [Yes]	No [No]
14. ¿Le ha golpeado con el pie? [14. Kicked you?]	1	2	3	4	Sí [Yes]	No [No]
15. ¿Ha intentado estrangularle? [15. Choked you?]	1	2	3	4	Sí [Yes]	No [No]
16. ¿Le ha amenazado con una pistola, un cuchillo o algo por el estilo? [16. Threatened to use or actually used a gun, knife or other weapon against you?]	1	2	3	4	Sí [Yes]	No [No]
17. ¿Le ha obligado a tener relaciones sexuales cuando no quería? [17. Forced you to have sexual intercourse when you did not want to?]	1	2	3	4	Sí [Yes]	No [No]
18. ¿Alguna vez tuvo relaciones sexuales cuando no quería porque tenía miedo de lo que podría hacerle? [18. Did you ever have sexual intercourse you did not want because you were afraid of what he might do?]	1	2	3	4	Sí [Yes]	No [No]

19. ¿Le ha obligado a hacer algo sexual que ha encontrado degradante o humillante? [19. Did he ever force you to do something sexual that you found degrading or humiliating?]	1	2	3	4	Sí [Yes]	No [No]
20. ¿Trató de evitar que viera a sus amigas/os? [20. Tries to keep you from seeing your friends?]	1	2	3	4	Sí [Yes]	No [No]
21. ¿Trató de restringirle el contacto con su familia? [21. Tries to restrict contact with your family of birth?]	1	2	3	4	Sí [Yes]	No [No]
22. ¿Insistió en conocer dónde estaba todo el tiempo? [22. Insists on knowing where you are at all times?]	1	2	3	4	Sí [Yes]	No [No]
23. ¿Le ignoró o le trató con indiferencia? [23. Ignores you or treats you indifferently?]	1	2	3	4	Sí [Yes]	No [No]
24. ¿Se enfadó si usted hablaba con otro/s hombre/s? [24. Gets angry if you speaks if you speak with another man?]	1	2	3	4	Sí [Yes]	No [No]
25. ¿Sospechaba a menudo de que usted le era infiel? [25. Is often suspicious that you are unfaithful?]	1	2	3	4	Sí [Yes]	No [No]
